# Loss of CLIC3 in CD8^+^ cytotoxic T lymphocytes is associated with immune dysfunction and poor prognosis in lung adenocarcinoma

**DOI:** 10.3389/fonc.2026.1975159

**Published:** 2026-09-30

**Authors:** Yinglong Li, Yidan Nie, Zhigang Han, Tingting Yu

**Affiliations:** 1 Department of Pulmonary Medicine, Affiliated Cancer Hospital of Xinjiang Medical University, Urumqi, Xinjiang, China; 2 Department of Urology, Affiliated Cancer Hospital of Xinjiang Medical University, Urumqi, Xinjiang, China

**Keywords:** CLIC3, cytotoxic T lymphocytes, lung adenocarcinoma, T cell exhaustion, tumor microenvironment

## Abstract

**Background:**

Lung adenocarcinoma (LUAD) is the most common histological subtype of lung cancer, with a dismal prognosis partly attributed to immune evasion mediated by T cell dysfunction within the tumor microenvironment. However, the molecular determinants governing CD8^+^ cytotoxic T lymphocyte functional competence remain incompletely understood. Chloride intracellular channel 3 (CLIC3) has been implicated in cancer progression, but its role in T cell immunity within the LUAD TME is unknown.

**Methods:**

We performed single-cell RNA sequencing on tumor tissues from nine LUAD patients spanning three pathological stages (adenocarcinoma *in situ* [AIS], minimally invasive adenocarcinoma [MIA], and invasive adenocarcinoma [IAC]), integrated with spatial transcriptomics, TCGA-LUAD bulk RNA-seq analysis, and flow cytometric validation in an independent cohort of 32 LUAD patients. Functional module scoring and pseudotime trajectory analysis were applied to characterize CLIC3-associated CTL functional states.

**Results:**

CLIC3 was predominantly expressed in CTLs and showed a stage-associated decline from AIS to MIA to IAC. CLIC3^+^ CTLs exhibited significantly higher module scores for cytotoxicity, cytoskeleton remodeling, interferon signaling, and chemotaxis compared to CLIC3- CTLs. Pseudotime trajectory analysis indicated that CLIC3^+^ cells were positioned at one end of the inferred transcriptional continuum, corresponding to a functionally active state, while CLIC3^-^ cells were positioned at the other, corresponding to a dysfunctional state, accompanied by a decline in CD247, GZMB, PRF1, and TNF along the trajectory. High CLIC3 expression in TCGA-LUAD was associated with significantly longer overall survival. Flow cytometry showed a stage-associated decrease in CLIC3 expression on CD8^+^ T cells with increasing tumor invasiveness and poor differentiation.

**Conclusions:**

CLIC3 serves as a novel marker of CTL functional competence in LUAD, and its stage-associated downregulation reflects T cell dysfunction during tumor progression. CLIC3 expression in CD8^+^ T cells may represent a predictive biomarker for immunotherapeutic response and a potential target for restoring T cell function in LUAD.

## 1 Introduction

Lung cancer remains the leading cause of cancer-related mortality worldwide, with lung adenocarcinoma (LUAD) being the most prevalent histological subtype, accounting for approximately 40% of all lung cancer cases (1, 2). Despite substantial advances in targeted therapy and immunotherapy over the past decade, the prognosis for LUAD patients, particularly those with advanced-stage disease, remains unsatisfactory, with a five-year overall survival rate of less than 30% (1). The intricate and heterogeneous nature of the tumor microenvironment (TME) has emerged as a critical determinant of tumor progression, therapeutic response, and patient outcome. Within the LUAD TME, the complex interplay between malignant cells and infiltrating immune cells, especially T lymphocytes, profoundly influences the trajectory of disease evolution (3). CD8^+^ cytotoxic T lymphocytes (CTLs) constitute the primary effector arm of the antitumor immune response, and their functional competence is essential for effective immune surveillance and tumor control (4). However, during tumor progression, CTLs frequently undergo a process of functional deterioration characterized by loss of effector functions, impaired T cell receptor (TCR) signaling, and upregulation of exhaustion markers, ultimately leading to immune evasion and tumor outgrowth (5). Elucidating the molecular determinants that govern CTL functional integrity within the LUAD microenvironment is therefore of paramount importance for developing novel immunotherapeutic strategies and identifying predictive biomarkers of treatment response.

The chloride intracellular channel (CLIC) protein family comprises six evolutionarily conserved, redox-sensitive members (CLIC1–CLIC6) that exist in both soluble cytosolic and membrane-associated forms (6). CLICs play diverse roles in cellular physiology, including ion channel activity, regulation of intracellular chloride homeostasis, endosomal trafficking, and angiogenesis (7). Accumulating evidence has implicated CLIC family members in tumorigenesis and cancer progression across multiple cancer types, including hepatocellular carcinoma, colorectal cancer, ovarian cancer, and lung cancer (8, 9). Among these, CLIC3 has recently emerged as a molecule of particular interest in oncology. CLIC3 functions as a glutathione-dependent oxidoreductase and has been shown to promote tumor cell proliferation, migration, and invasion through the inhibition of transglutaminase 2 (TGM2) and modulation of intracellular chloride and reactive oxygen species (ROS) levels (10). In lung cancer, recent studies have demonstrated that CLIC3 is significantly upregulated in tumor tissues and that its high expression is associated with poor patient prognosis (11, 12). However, the expression pattern and functional significance of CLIC3 specifically within the T cell compartment of the LUAD microenvironment remain largely unexplored. Given the distinct expression profiles of CLIC isoforms across different lung cell types and the emerging role of CLIC family members in immune modulation (13), we hypothesized that CLIC3 may serve as a critical marker of CTL functional states within the LUAD tumor microenvironment.

In this study, we employed an integrative approach combining single-cell RNA sequencing (scRNA-seq) of nine LUAD samples spanning three pathological stages (adenocarcinoma *in situ* [AIS], minimally invasive adenocarcinoma [MIA], and invasive adenocarcinoma [IAC]), with spatial transcriptomics, bulk RNA-seq analysis from the TCGA-LUAD cohort (n = 517), and flow cytometric validation in an independent cohort of 32 LUAD patients. Our findings reveal that CLIC3 is predominantly expressed in CTLs and is downregulated with increasing tumor invasiveness. CLIC3^+^ CTLs exhibit enhanced functional module activities, including cytotoxicity, cytoskeletal remodeling, interferon signaling, and chemotaxis, and are positioned at the early stage of the CTL differentiation trajectory. Mechanistically, the loss of CLIC3 is accompanied by the decline of TCR signaling components and effector molecules, consistent with the acquisition of an exhausted phenotype. Collectively, our results identify CLIC3 as a novel marker of CTL functional competence and suggest that its downregulation represents a critical event in the immune dysfunction associated with LUAD progression, potentially offering new insights for immunotherapeutic intervention.

## 2 Materials and methods

### 2.1 Patient enrollment

A total of 32 patients with primary lung cancer who underwent surgical resection at Affiliated Cancer Hospital of Xinjiang Medical University, between February 2025 and June 2025 were enrolled in this study. Peripheral blood samples were collected from all patients prior to any therapeutic intervention. Comprehensive clinicopathological data, including pathological type, tumor stage, and histological subtype, were meticulously documented based on the postoperative pathological reports. All patients were diagnosed according to the Guidelines for the Diagnosis and Treatment of Lung Cancer (2024 Edition) of the Chinese Society of Clinical Oncology (CSCO). This was a cross-sectional study, and no longitudinal follow-up data regarding patient survival or disease recurrence were collected. The study protocol was approved by the Institutional Review Board of Affiliated Cancer Hospital of Xinjiang Medical University, and written informed consent was obtained from each participant prior to enrollment.

### 2.2 Data acquisition

Transcriptomic data for this study were obtained from multiple complementary sources. Bulk RNA-sequencing data and corresponding clinical annotations for 517 LUAD patients were retrieved from The Cancer Genome Atlas (TCGA) database (https://portal.gdc.cancer.gov/), comprising RNA-seq profiles processed through the standard TCGA pipeline, with raw count data downloaded for subsequent analyses. Single-cell RNA-sequencing data were acquired from the Gene Expression Omnibus (GEO) repository under accession number GSE189357, which includes scRNA-seq profiles from nine resected lung tissue samples (TD1-9, three each from AIS, MIA and IAC cases) obtained from nine treatment-naïve patients. Spatial transcriptomics data were obtained from the GEO repository under accession number GSE189487, comprising six paired samples from the aforementioned patients subjected to spatial transcriptomics (ST) analysis. All data processing and analytical workflows were implemented in R (version 4.2.0) with appropriate computational biology packages.

### 2.3 Single-cell RNA sequencing analysis

Single-cell RNA sequencing data analysis was conducted using Seurat (version 5.0.0) and complementary R packages including dplyr (1.1.4), patchwork (1.2.0), and tidyverse (2.0.0). The analytical pipeline incorporated quality control based on mitochondrial gene content (<20%) and unique molecular identifier (UMI) counts (200-6,000 per cell), followed by data normalization using the “LogNormalize” method with a scale factor of 10,000. Highly variable genes were identified using the variance-stabilizing transformation (vst) method, and principal component analysis (PCA) was performed for dimensional reduction. Cell clustering was achieved using the FindNeighbors and FindClusters functions with a resolution parameter of 0.5, and results were visualized through T-distributed stochastic neighbor embedding (TSNE). Cell type annotation was performed according to established lineage-specific markers, curated with the aid of the CellMarker 2.0 database (https://bio-bigdata.hrbmu.edu.cn/CellMarker2.0/). Differential gene expression analysis between cell clusters or experimental conditions was performed using the Wilcoxon rank-sum test with thresholds of minimum percentage (min.pct = 0.1) and log-fold change (logfc.threshold = 0.25). Genes with an adjusted P-value < 0.05 were considered significantly differentially expressed.

Pseudotime trajectory analysis was performed using Slingshot (version 2.10.0) to infer the developmental trajectory of cytotoxic T lymphocytes. The analysis was based on Uniform Manifold Approximation and Projection (UMAP) dimensional reduction coordinates, with cluster assignments derived from Seurat clustering results. Pseudotime values were computed for each cell, and gene expression dynamics along the pseudotime trajectory were assessed using generalized additive models (GAMs) with the loess smoothing method.

### 2.4 Functional enrichment analysis

Functional enrichment analysis was performed using the Database for Annotation, Visualization, and Integrated Discovery (DAVID, version 2023q4). Differentially expressed genes (DEGs) identified between CLIC3^+^ and CLIC3^-^ cytotoxic T lymphocytes from single-cell RNA sequencing data were submitted to DAVID for Gene Ontology (GO) biological process enrichment and Kyoto Encyclopedia of Genes and Genomes (KEGG) pathway analysis. Additionally, to further validate the functional relevance of CLIC3 at the bulk transcriptomic level, we performed differential expression analysis on the TCGA LUAD dataset based on CLIC3 expression levels. Patients were stratified into CLIC3-high and CLIC3-low groups according to the median CLIC3 expression, and DEGs between the two groups were subjected to GO biological process enrichment and KEGG pathway analysis using DAVID. Enrichment results from both analyses were filtered with a significance threshold of adjusted P-value < 0.05, and the top significantly enriched terms were ranked by ascending adjusted P-value for reporting.

### 2.5 Bioinformatic analysis of CLIC3 functional associations in cytotoxic T lymphocytes

Functional module scoring analysis was performed to characterize the functional states of T cell subsets. Module scores for predefined gene sets representing key T cell functions were calculated, including cytotoxicity (GZMA, GZMB, GZMH, GZMK, GZMM, PRF1, GNLY, NKG7, KLRF1, KLRB1), cytoskeleton remodeling (ACTB, ACTG1, TUBA1B, TUBB, WAS, WASL, ARPC2, ARPC3, RAC1, CDC42, EZR, MSN, VCL, FLNA, SPTBN1), interferon (IFN) signaling (IFNG, IFNGR1, IFNGR2, STAT1, STAT4, IRF1, IRF8, CXCL9, CXCL10, CXCL11), and chemotaxis/migration (CCL3, CCL4, CCL5, CCL4L2, XCL1, XCL2, CXCR3, CCR5, CCR7, SELL). Module scores for each functional category were calculated using the AddModuleScore function in Seurat with parameters set at min.pct = 0.1, logfc.threshold = 0.25, and 100 control genes matched for expression level. Differential module scores between CLIC3^+^ and CLIC3^-^ CTLs were evaluated using the Wilcoxon rank-sum test with significance set at p < 0.05. The correlation between CLIC3 expression and functional module scores was assessed using Spearman’s rank correlation coefficient. All analyses were performed in R (version 4.2.0) using Seurat (4.3.0), ggplot2 (3.4.0), and dplyr (1.1.0) packages, with multiple testing correction applied using the Benjamini-Hochberg method where appropriate.

### 2.6 Pseudotime trajectory analysis of CLIC3^+^ cytotoxic T lymphocytes

Pseudotime trajectory analysis was performed using Slingshot (version 2.10.0) to infer a transcriptional continuum of CTLs. Because the scRNA-seq dataset represents a cross-sectional snapshot of cells rather than a true time series, the resulting ordering is a computational construct based on transcriptional similarity and does not represent a directly measured temporal sequence. The analysis was based on UMAP dimensional reduction coordinates derived from the Seurat clustering pipeline, with cluster assignments serving as inputs for lineage construction. The trajectory was initialized with a starting cluster determined by the highest proportion of CLIC3^+^ cells, and the principal curve algorithm was applied to fit smooth branching trajectories through the dimensional reduction space. Pseudotime values were subsequently computed for each cell along the inferred lineages, representing the relative position of cells along the transcriptional continuum rather than their true temporal progression. To characterize the molecular dynamics associated with CLIC3 expression, we assessed gene expression trends along the inferred pseudotime trajectory using GAMs with loess smoothing (span = 0.3). The distribution of CLIC3^+^ and CLIC3^-^ cells along pseudotime was compared using the Wilcoxon rank-sum test, and the proportion of CLIC3^+^ cells across pseudotime stages was quantified. Additionally, the expression dynamics of key functional genes, including CD247, GZMB, PRF1, TOX, TNF, and LAT, were evaluated along the trajectory to delineate the transcriptional transition associated with CLIC3 loss. All pseudotime analyses were performed in R (version 4.2.0) using Slingshot (2.10.0), ggplot2 (3.4.0), and tradeSeq (1.10.0) where applicable.

### 2.7 Flow cytometry

Peripheral blood samples were collected from all enrolled patients into heparinized tubes prior to any therapeutic intervention. Peripheral blood mononuclear cells (PBMCs) were isolated by Ficoll-Paque density gradient centrifugation (GE Healthcare, USA) within 4 hours of collection. For surface staining, PBMCs were incubated with fluorochrome-conjugated antibodies against human CD3 (BD, USA) and CD8 (BD, USA) for 30 minutes at 4 °C in the dark. Following surface staining, cells were fixed and permeabilized using the BD Transcription Factor Buffer Set (BD, USA) according to the manufacturer’s protocol. Briefly, cells were fixed with 1× Fix/Perm buffer (containing 5.3% formaldehyde) for 40–50 minutes at 4 °C, then washed and permeabilized with 1× Perm/Wash buffer. For intracellular detection of CLIC3, cells were incubated with a mouse anti-human CLIC3 monoclonal antibody (Abnova, Taiwan, China) at 4 °C for 40–50 minutes. After washing, cells were further incubated with a fluorescently labeled secondary antibody (Thermo Fisher Scientific, USA) for 30 minutes at 4 °C in the dark. Appropriate isotype-matched control antibodies (BD, USA) and fluorescence-minus-one (FMO) controls were included in each experiment to establish gating strategies and confirm staining specificity. Following staining, cells were resuspended in staining buffer and acquired on a flow cytometer (Canto II)(BD, USA). Data analysis was performed using FlowJo software (BD, USA). The gating strategy was as follows: lymphocytes were first identified based on forward scatter (FSC) and side scatter (SSC) characteristics, and then CD3^+^CD8^+^ T cells were gated. CLIC3 expression was evaluated within the CD3^+^CD8^+^ T cell population, and the percentage of CLIC3^+^ cells was quantified.

### 2.8 Statistical analysis

Statistical analyses were performed using R (version 4.2.0) and GraphPad Prism (version 9.5.0, GraphPad Software, USA). For comparisons between two groups, the unpaired two-tailed Student’s t-test was applied for normally distributed continuous variables, while the Mann-Whitney U test was used for non-normally distributed variables. For comparisons among three or more groups (e.g., AIS, MIA, and IAC), one-way analysis of variance (ANOVA) followed by Tukey’s *post-hoc* test was used for normally distributed data, and the Kruskal-Wallis test followed by Dunn’s *post-hoc* test was applied for non-normally distributed data. Categorical variables were compared using Pearson’s χ² test or Fisher’s exact test, as appropriate. Correlation analyses were performed using Spearman’s rank correlation coefficient. Differential gene expression analysis in single-cell RNA sequencing data was performed using the Wilcoxon rank-sum test with Benjamini-Hochberg correction for multiple testing. All statistical tests were two-sided, and a P value < 0.05 was considered statistically significant. Data are presented as mean ± standard deviation (SD), as appropriate based on data distribution.

## 3 Result

### 3.1 Single-cell transcriptomic landscape of lung adenocarcinoma progression

To delineate the cellular heterogeneity underlying the progression from AIS to MIA and IAC, we performed scRNA-seq on tumor tissues from nine treatment-naïve patients (three per pathological stage), complemented by bulk RNA-seq analysis from the TCGA-LUAD cohort (**Figure 1A**).

**Figure 1:**
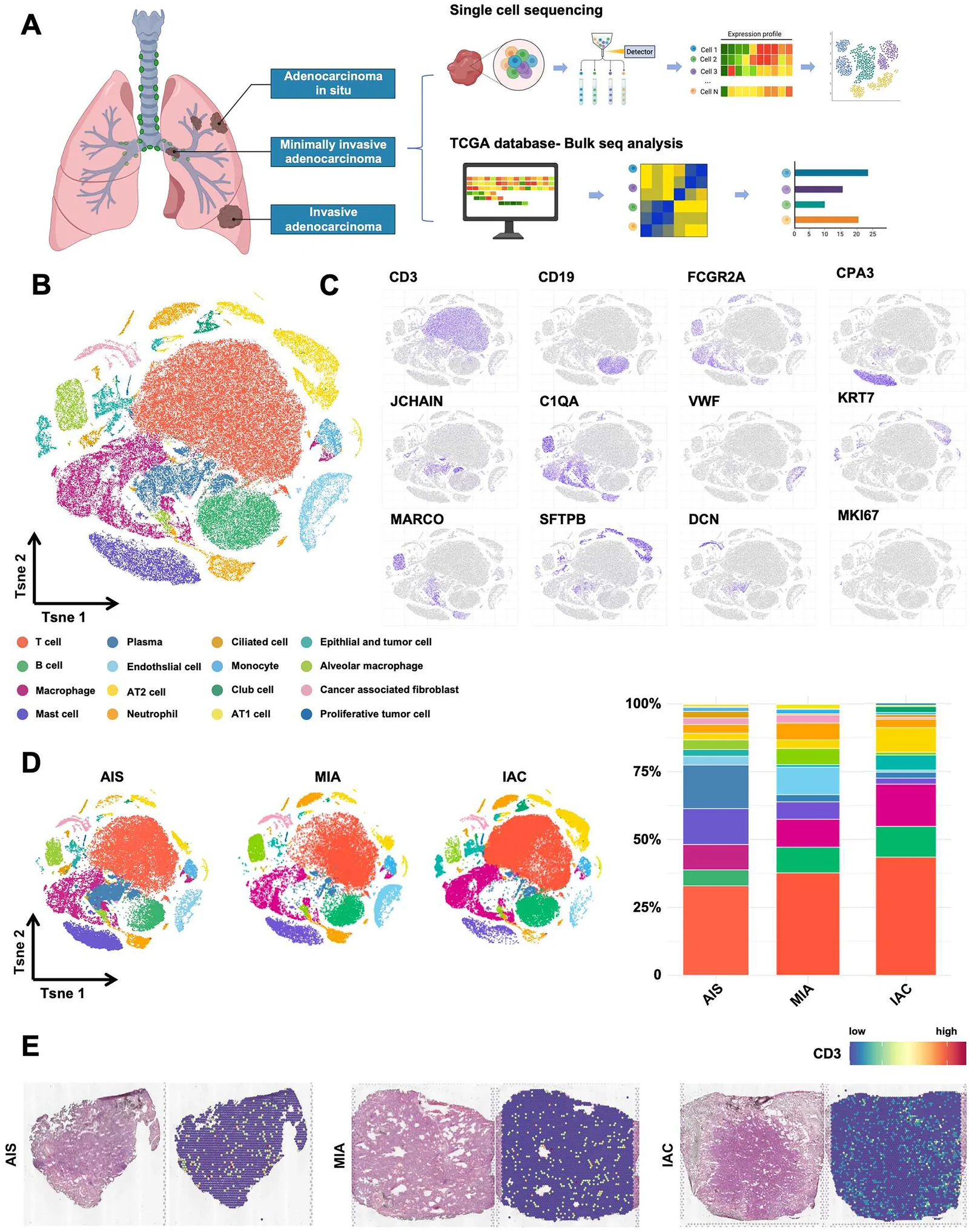
Single-cell transcriptomic landscape and tumor microenvironment composition across lung adenocarcinoma progression. **(A)** Schematic overview of the study design. Single-cell RNA sequencing (scRNA-seq) was performed on tumor tissues from AIS, MIA, and IAC patients (n = 3 per group), integrated with bulk RNA-seq analysis from the TCGA-LUAD cohort. **(B)** t-SNE projection of 47,647 cells from all samples, colored by cell type annotation. Sixteen distinct cell lineages were identified based on transcriptional profiles. **(C)** Dot plot showing the expression of representative lineage-specific marker genes across the annotated cell types. Dot size represents the percentage of cells expressing the gene, and color intensity indicates the average expression level. **(D)** Bar plot displaying the relative proportion of each cell type within AIS, MIA, and IAC samples. T cells comprised the dominant immune population and exhibited an increase from AIS (32.9%) to MIA (37.7%) and IAC (43.4%). **(E)** Spatial transcriptomic mapping of CD3 expression in representative tissue sections from AIS, MIA, and IAC samples. Spatial CD3 signal intensity increased with disease stage, corroborating the scRNA-seq findings at the tissue level.

Unsupervised clustering of the scRNA-seq data identified 16 distinct cell clusters, which were visualized using t-SNE (**Figure 1B**). These clusters were annotated into major cell lineages based on established marker gene expression, including T cells, B cells, plasma cells, macrophages, monocytes, dendritic cells, neutrophils, mast cells, endothelial cells, fibroblasts, alveolar epithelial cells, club cells, ciliated cells, and proliferative tumor cells. The specificity of these annotations was confirmed by the distinct expression patterns of representative lineage-specific markers across cell types (**Figure 1C**).

To investigate the compositional dynamics of the tumor microenvironment TME across disease stages, we compared the relative proportions of each cell type among AIS, MIA, and IAC samples (**Figure 1D**). Notably, T cells constituted the predominant immune population in all stages. Interestingly, the proportion of T cells increased from AIS (32.9%) to MIA (37.7%) and further to IAC (43.4%), suggesting an enhanced immune infiltration in more aggressive tumors (**Supplementary Table 1**).

To validate this observation at the spatial level, we analyzed spatial transcriptomics data from six paired samples (two per stage). Consistent with the scRNA-seq findings, spatial mapping of CD3 expression revealed a gradual increase in T cell abundance from AIS to IAC (**Figure 1E**), confirming the elevated T cell infiltration in invasive tumors at the tissue architecture level.

### 3.2 Identification of CLIC3 as a key gene associated with cytotoxic T lymphocyte dysfunction in lung adenocarcinoma progression

To identify key molecular alterations in T cells during lung adenocarcinoma progression, we performed differential expression analysis on T cells isolated from the scRNA-seq data. A total of 2,473 differentially expressed genes were identified between AIS and MIA, and 3,146 DEGs between MIA and IAC. Among these, 1,133 genes were commonly dysregulated across both comparisons. Notably, among the overlapping DEGs, 14 genes exhibited a concordant direction of change, with 7 genes consistently downregulated (LINC01871, CEBPD, ANKRD28, SPON2, HNRNPUL1, CLIC3, and SYTL3) (**Figure 2A**). This concordant pattern of downregulation across both comparisons suggested that these genes might play critical roles in T cell dysfunction associated with increasing tumor aggressiveness. Among these seven candidates, CLIC3 was selected for further investigation for several reasons. First, among the seven candidates, CLIC3 has the most clearly defined molecular function in the context of ion transport and redox regulation, namely chloride channel activity and glutathione-dependent oxidoreductase activity, which provides a tractable basis for mechanistic investigation. Second, members of the CLIC family have been increasingly implicated in immune cell function and tumor-immune interactions [ref], whereas the other six genes have been predominantly studied in contexts unrelated to T cell immunity, with limited evidence for a role in CTL function. Third, CLIC3 was predominantly expressed in the CTL subset and exhibited the clearest stage-associated decline in our scRNA-seq data, making it the most promising candidate for further characterization.

**Figure 2:**
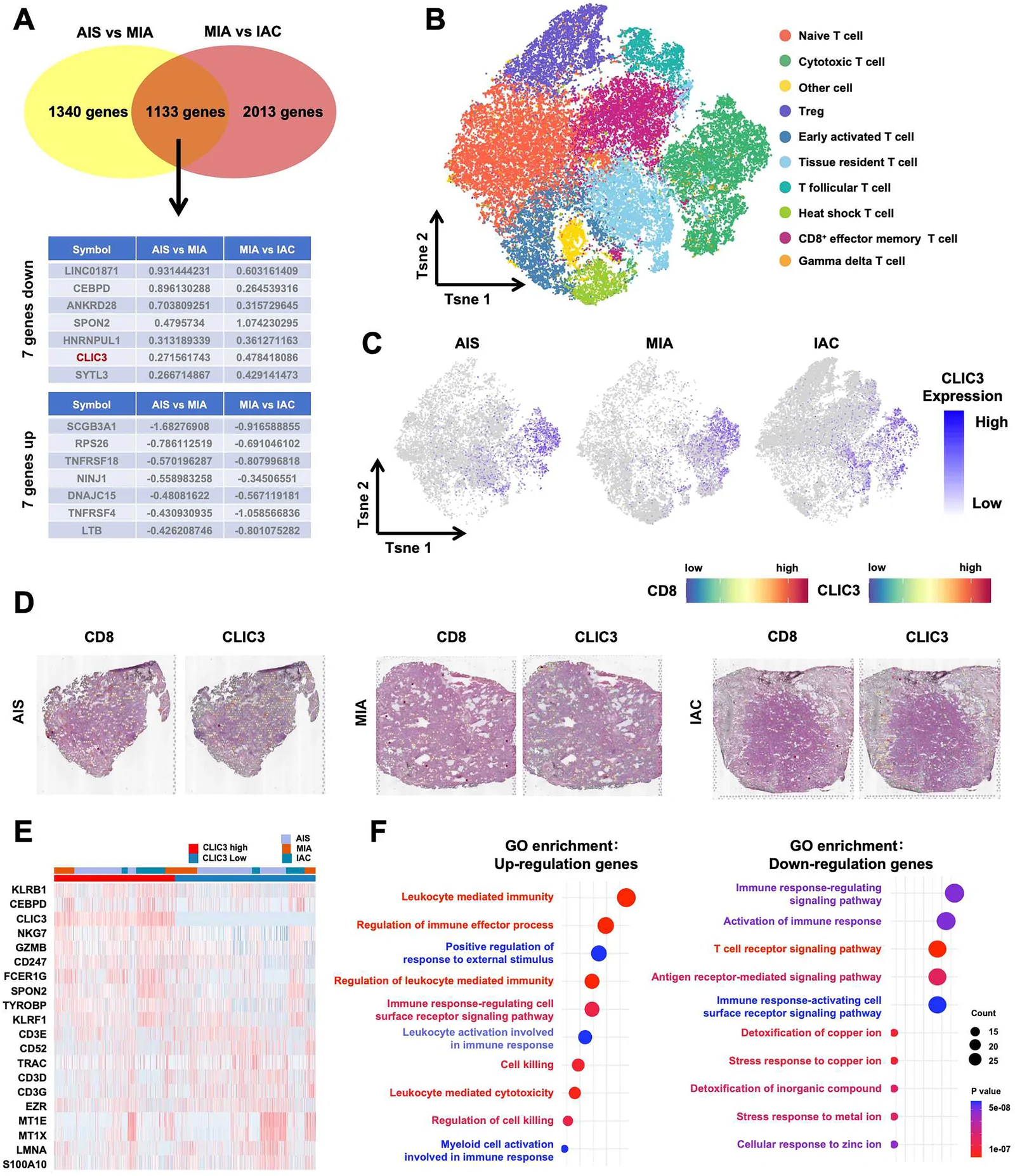
CLIC3 is downregulated in cytotoxic T lymphocytes during lung adenocarcinoma progression. **(A)** Differential expression analysis of T cells between AIS vs MIA and MIA vs IAC. Up: Venn diagram showing the number of DEGs in each comparison and their overlaps. Down: Table of the genes consistently downregulated and upregulated across both comparisons. **(B)** t-SNE projection of T cell subclusters from scRNA-seq data, revealing 10 distinct T cell subsets. **(C)** Violin plots showing CLIC3 expression across pathological stages (AIS, MIA, IAC) in different T cell subsets. CLIC3 is predominantly expressed in cytotoxic T lymphocytes (CTLs) and decreases from AIS to IAC. **(D)** Spatial transcriptomic mapping of CD8 and CLIC3 expression in representative tissue sections from AIS, MIA, and IAC samples. CLIC3 signal decreases with disease progression, consistent with the scRNA-seq findings. **(E)** Heatmap showing differentially expressed genes between CLIC3+ and CLIC3- CTLs. Genes positively correlated with CLIC3 include KLRB1, CEBPD, NKG7, GZMB, and CD247; negatively correlated genes include CD3E, CD3D, CD52, and TRAC. **(F)** Gene Ontology (GO) enrichment analysis of DEGs between CLIC3+ and CLIC3- CTLs. Upregulated genes in CLIC3+ CTLs are enriched in immune effector processes, while downregulated genes are enriched in TCR signaling and immune response pathways.

Subclustering of T cells revealed 10 distinct T cell subsets, which were visualized using T-SNE projection (**Figure 2B**). We next examined CLIC3 expression across the three pathological stages. CLIC3 was predominantly expressed in the CTL subset, and its expression levels decreased from AIS to MIA to IAC, suggesting a stage-dependent loss of CLIC3 in CTLs during tumor progression (**Figure 2C**). To further examine this observation at the spatial level, we analyzed spatial transcriptomics data for CD8 and CLIC3 expression. Consistent with the broad expression of CLIC3 across multiple lung cell types, CLIC3 signal did not completely overlap with CD8, indicating that CLIC3 is enriched in, rather than specific to, CTLs. Nevertheless, we observed a clear reduction in CLIC3 signal from AIS to IAC, which was spatially consistent with the scRNA-seq findings. (**Figure 2D**). To characterize the transcriptional programs associated with CLIC3 expression in CTLs, we performed differential expression analysis between CLIC3^+^ and CLIC3^-^ CTLs (**Figure 2E**). Genes positively correlated with CLIC3 included KLRB1, CEBPD, NKG7, GZMB, and CD247, while negatively correlated genes included CD3E, CD3D, CD52, and TRAC. To provide a functional annotation of these DEGs, we performed GO enrichment analysis (**Figure 2F**). Genes upregulated in CLIC3^+^ CTLs were annotated to immune-related biological processes such as leukocyte mediated immunity, regulation of immune effector process, and positive regulation of response to external stimulus, whereas genes downregulated in CLIC3^+^ CTLs were annotated to immune response-regulating signaling pathway, activation of immune response, and T cell receptor signaling pathway. As these categories largely reflect the transcriptional differences defined by CLIC3 stratification itself, this analysis serves primarily to provide biological context for the subsequent module scoring and pseudotime analyses rather than to constitute an independent finding.

These findings indicate that CLIC3 expression marks a CTL population with enhanced effector functions, while CLIC3 loss is associated with impaired TCR signaling and immune response activation.

### 3.3 CLIC3^+^ cytotoxic T lymphocytes exhibit enhanced functional module activity along an inferred transcriptional continuum

To further characterize the functional significance of CLIC3 expression in CTLs, we performed module scoring analysis using predefined gene sets representing four key T cell functions: cytotoxicity, cytoskeleton remodeling, IFN signaling, and chemotaxis. Compared with CLIC3^-^ CTLs, CLIC3^+^ CTLs exhibited significantly higher module scores across all four functional categories (**Figure 3A**), indicating that CLIC3 expression marks CTLs with superior effector capacity, cytoskeletal integrity, IFN responsiveness, and migratory potential.

**Figure 3:**
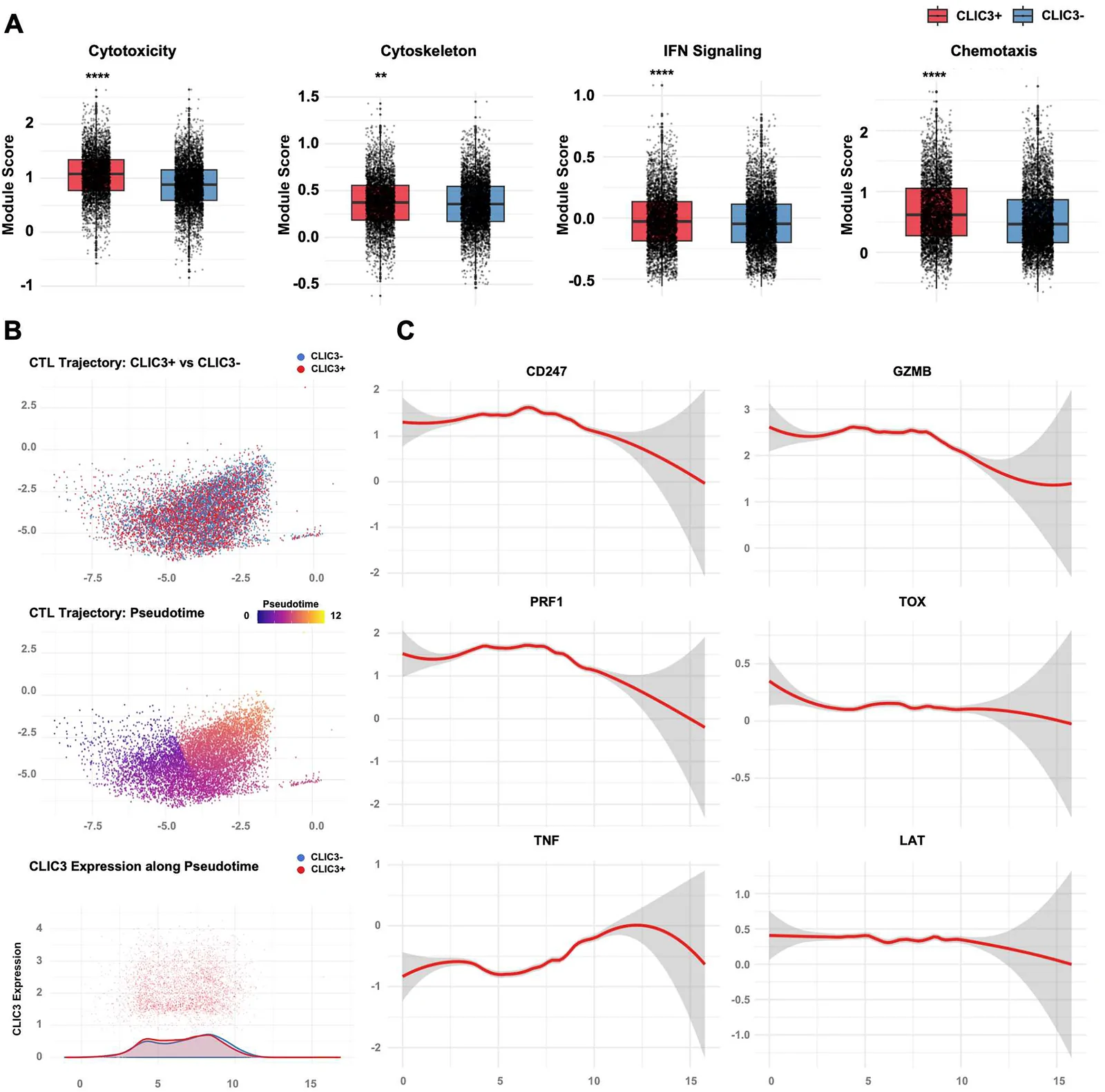
CLIC3^+^ CTLs exhibit enhanced functional module activity and are positioned at the early stage of the differentiation trajectory. **(A)** Module scoring analysis of CTLs stratified by CLIC3 expression. Box plots show the module scores for cytotoxicity, cytoskeleton, IFN signaling, and chemotaxis in CLIC3^+^ (red) versus CLIC3^-^ (blue) CTLs. CLIC3^+^ CTLs demonstrate significantly higher scores across all four functional categories (Wilcoxon rank-sum test). **(B)** Pseudotime trajectory analysis of CTLs. CLIC3^+^ cells (red) are predominantly localized at the early phase of the trajectory, while CLIC3^-^ cells (blue) accumulate at the late phase. **(C)** Expression dynamics of six key functional genes along pseudotime. CD247 (TCR signaling), GZMB and PRF1 (cytotoxicity), TNF (pro-inflammatory cytokine), LAT (synapse signaling), and TOX (exhaustion-associated transcription factor) all exhibit decline along the pseudotime trajectory, mirroring the downregulation pattern of CLIC3. **P<0.01;****P<0.0001.

To investigate the transcriptional relationship between CLIC3^+^ and CLIC3^-^ CTLs, we performed pseudotime trajectory analysis using Slingshot. Given that the dataset is cross-sectional, this analysis provides an inferred ordering of cells along a transcriptional continuum rather than a measured temporal sequence. The inferred trajectory placed CLIC3^+^ cells predominantly at one end of the continuum and CLIC3- cells at the other, consistent with a transition from a more functional to a less functional state (**Figure 3B**). This distribution pattern suggests that CLIC3 expression is associated with a functionally active CTL state, whereas CLIC3 loss is associated with a state characterized by reduced effector features.

We next examined the expression dynamics of six key functional genes along the inferred trajectory: CD247 (TCR signaling component), GZMB and PRF1 (cytotoxic effector molecules), TNF (pro-inflammatory cytokine), LAT (synapse signaling adaptor), and TOX (exhaustion-associated transcription factor). CD247, GZMB, PRF1, and TNF exhibited a decline along the trajectory, paralleling the distribution pattern of CLIC3. In contrast, LAT showed an initial decrease followed by a relatively stable plateau at later positions along the trajectory, while TOX expression remained relatively constant (**Figure 3C**). This coordinated decline of effector- and signaling-related genes along with CLIC3 suggests that CLIC3 loss is part of a broader transcriptional program associated with reduced effector function, TCR signaling capacity, and synaptic integrity. Notably, the parallel decline of these functionally diverse genes along with CLIC3 further supports the role of CLIC3 as a marker associated with CTL functional competence.

### 3.4 Clinical relevance and prognostic value of CLIC3 expression in lung adenocarcinoma

To evaluate the clinical significance of CLIC3 expression across cancer types, we first examined its expression patterns in tumor versus adjacent normal tissues with TCGA pan-cancer dataset. CLIC3 expression exhibited distinct and cancer type-specific dysregulation patterns (**Figure 4A**). In certain cancer types, such as bladder urothelial carcinoma (BLCA) and cervical squamous cell carcinoma (CESC), CLIC3 was significantly upregulated in tumors compared with adjacent normal tissues. Conversely, in other cancer types, including LUAD, lung squamous cell carcinoma (LUSC), and head and neck squamous cell carcinoma (HNSC), CLIC3 expression was significantly lower in tumor tissues than in adjacent normal counterparts. This cancer type-specific expression pattern suggests that the role of CLIC3 may vary depending on the tumor microenvironment and tissue context.

**Figure 4:**
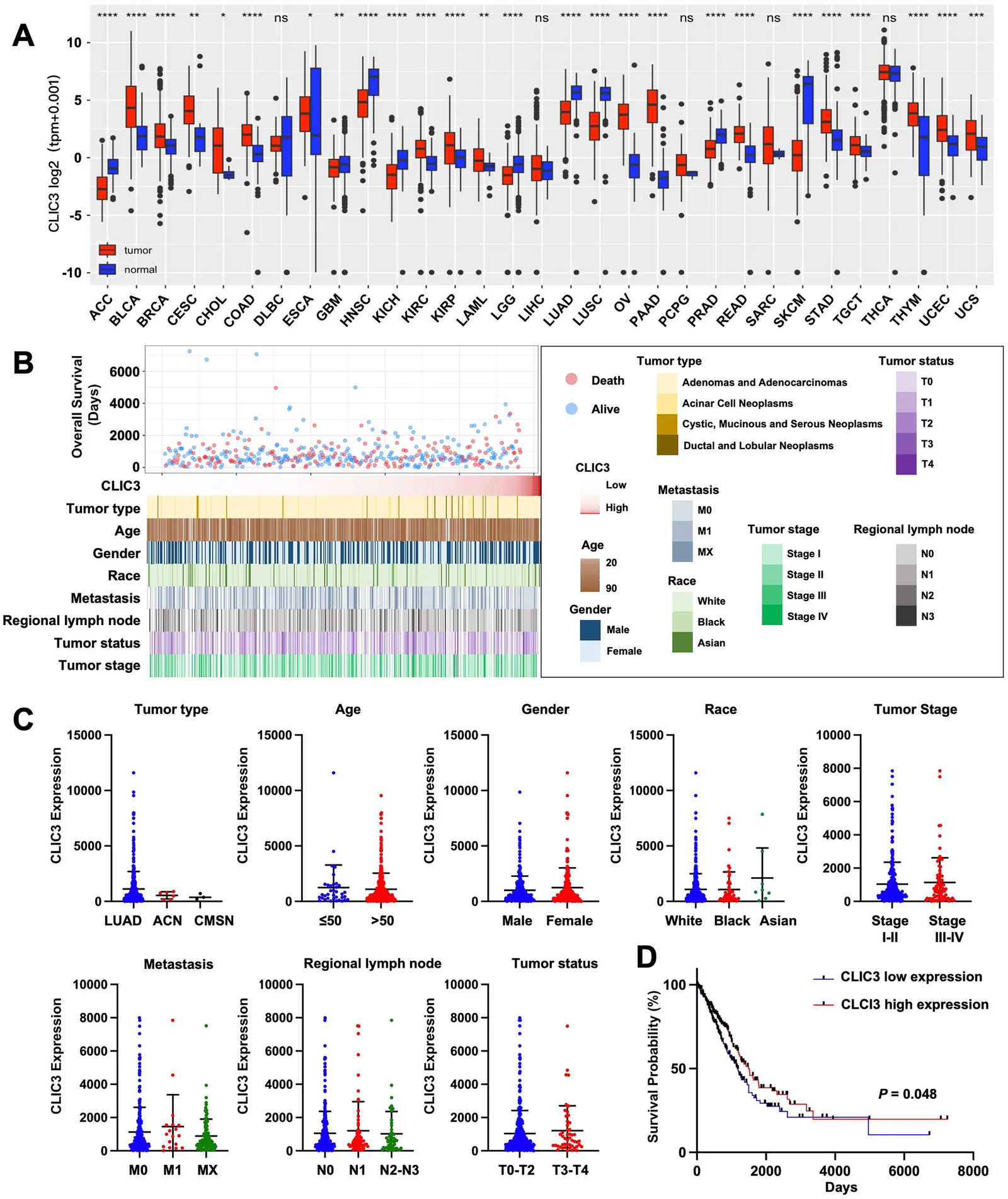
Clinical relevance and prognostic value of CLIC3 expression in lung adenocarcinoma. **(A)** Pan-cancer analysis of CLIC3 expression in tumor versus adjacent normal tissues from TCGA datasets. CLIC3 exhibits cancer type-specific dysregulation patterns. **(B)** Overview of the TCGA-LUAD cohort clinicopathological features analyzed in this study. **(C)** CLIC3 expression stratified by TCGA disease type annotations, including LUAD and samples annotated as “Acinar Cell Neoplasms” (ACN) and “Cystic, Mucinous and Serous Neoplasms”(CMSN), and by various clinicopathological parameters within the TCGA-LUAD cohort, including age (≤50 vs. >50), gender (male vs. female), race (White, Black, Asian), pathological stage (Stage I–II vs. Stage III–IV), T stage (T0–T2 vs. T3–T4), regional lymph node status (N0 vs. N1 vs. N2–N3), distant metastasis (M0 vs. M1 vs. MX), and tumor status (T0–T2 vs. T3–T4). No significant associations were observed between CLIC3 expression and these clinical parameters (all P > 0.05). **(D)** Kaplan–Meier survival analysis comparing overall survival between CLIC3-high and CLIC3-low LUAD patients. High CLIC3 expression is significantly associated with prolonged overall survival, suggesting its potential role as a favorable prognostic biomarker. *P<0.05;**P<0.01;***P<0.001;****P<0.0001.

We next investigated the association between CLIC3 expression and clinicopathological features in the TCGA-LUAD cohort (**Figure 4B**). The distribution of CLIC3 expression was examined across various clinical parameters, including age, gender, race, pathological stage, tumor status, lymph node metastasis, and distant metastasis (**Figure 4C**). However, no significant associations were observed between CLIC3 expression levels and any of the clinicopathological features examined. Despite the lack of association with conventional clinicopathological parameters, Kaplan–Meier survival analysis revealed that patients with high CLIC3 expression had significantly longer overall survival compared with those with low CLIC3 expression (**Figure 4D**). This prognostic advantage suggests that CLIC3 expression may serve as a favorable prognostic biomarker in LUAD, potentially reflecting the presence of functionally competent T cells within the tumor microenvironment.

### 3.5 Tissue expression landscape and subcellular localization of CLIC3 in the lung tissue

To further characterize CLIC3 expression beyond the immune context, we performed GO and KEGG enrichment analyses using CLIC3-correlated genes from the TCGA-LUAD cohort. GO biological process (BP) analysis revealed enrichment in pathways related to angiogenesis, fatty acid beta-oxidation, cytoplasmic translation, regulation of actin cytoskeleton organization, and transforming growth factor beta receptor signaling pathway. GO cellular component (CC) analysis showed enrichment in cytosol, extracellular exosome, cytoplasm, mitochondrion, focal adhesion, vesicle, peroxisome, and endosome membrane. GO molecular function (MF) analysis revealed enrichment in protein binding, oxidoreductase activity, identical protein binding, ubiquitin protein ligase binding, molecular adaptor activity. KEGG pathway analysis identified enrichment in pathways related to metabolic pathways, endocytosis, peroxisome, (**Figure 5A**). Notably, these enriched pathways were largely associated with general cellular metabolism, structural processes, and subcellular organelle functions rather than immune-related functions, consistent with the multi-cellular expression pattern of CLIC3 in the lung.

**Figure 5:**
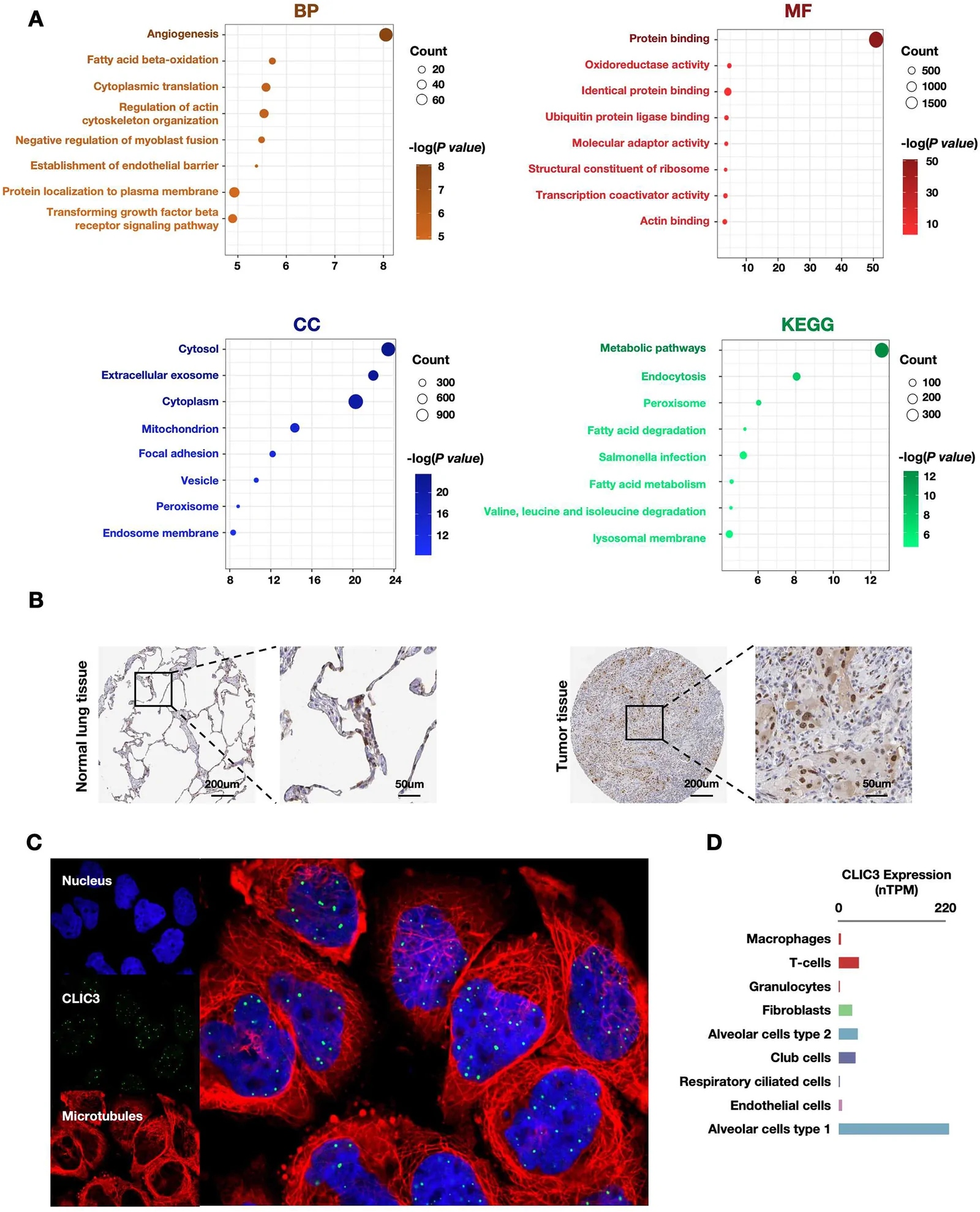
Tissue expression landscape and subcellular localization of CLIC3 in the lung. **(A)** Gene Ontology (GO) and Kyoto Encyclopedia of Genes and Genomes (KEGG) enrichment analyses of CLIC3-correlated genes in the TCGA-LUAD cohort. **(B)** Immunohistochemistry (IHC) staining of CLIC3 in normal lung tissue from the Human Protein Atlas (https://v21.proteinatlas.org). CLIC3 exhibits predominantly nuclear localization with a punctate distribution pattern. **(C)** Multiplex immunofluorescence (mIF) staining of CLIC3 in lung tissue confirming its punctate nuclear distribution, resembling the pattern of nuclear bodies or splicing speckles. **(D)** Cell type-specific expression of CLIC3 in the human lung based on Human Protein Atlas single-cell data. CLIC3 is broadly expressed across multiple cell types, with highest expression in alveolar cells type, followed by T cells and fibroblasts. nTPM, normalized transcripts per million.

To investigate the spatial and cellular distribution of CLIC3 protein in lung tissue, we examined immunohistochemistry (IHC) images from the Human Protein Atlas https://v21.proteinatlas.org). CLIC3 staining was predominantly localized in the nucleus, with a distinct punctate pattern, suggesting potential association with nuclear speckles or subnuclear structures (**Figure 5B**). This nuclear localization was further confirmed by multiplex immunofluorescence (mIF) staining, which revealed that CLIC3 exhibited a punctate distribution within the nucleus, resembling the typical pattern of nuclear bodies or splicing speckles (**Figure 5C**). Notably, CLIC3 expression was not limited to immune cells; single-cell resolution expression profiling from the Human Protein Atlas revealed that CLIC3 was expressed in multiple cell types in the lung, with the highest expression observed in alveolar cells type, followed by T cells and fibroblasts (**Figure 5D**). This broad cellular distribution, particularly the abundant expression in alveolar epithelial cells, likely explains the relatively limited enrichment of immune-related pathways in the TCGA-based functional analysis, as bulk transcriptomic data reflect the composite signal from both immune and non-immune cell populations.

### 3.6 Flow cytometric analysis of CLIC3 expression on peripheral blood CD8^+^ T cells from lung adenocarcinoma patients

To examine whether CLIC3 expression on circulating T cells shows a similar trend to that observed in tumor-infiltrating T cells, we performed flow cytometric analysis of CLIC3 expression on peripheral blood CD8^+^ T cells from 32 lung adenocarcinoma patients across three pathological stages (AIS, MIA, and IAC). The gating strategy was as follows: lymphocytes were first identified based on FSC and SSC characteristics, followed by selection of CD3^+^CD8^+^ T cells, and finally CLIC3^+^ cells were gated to determine the percentage of CLIC3 expressing cells within the CD8^+^ T cell population (**Figure 6A**).

**Figure 6:**
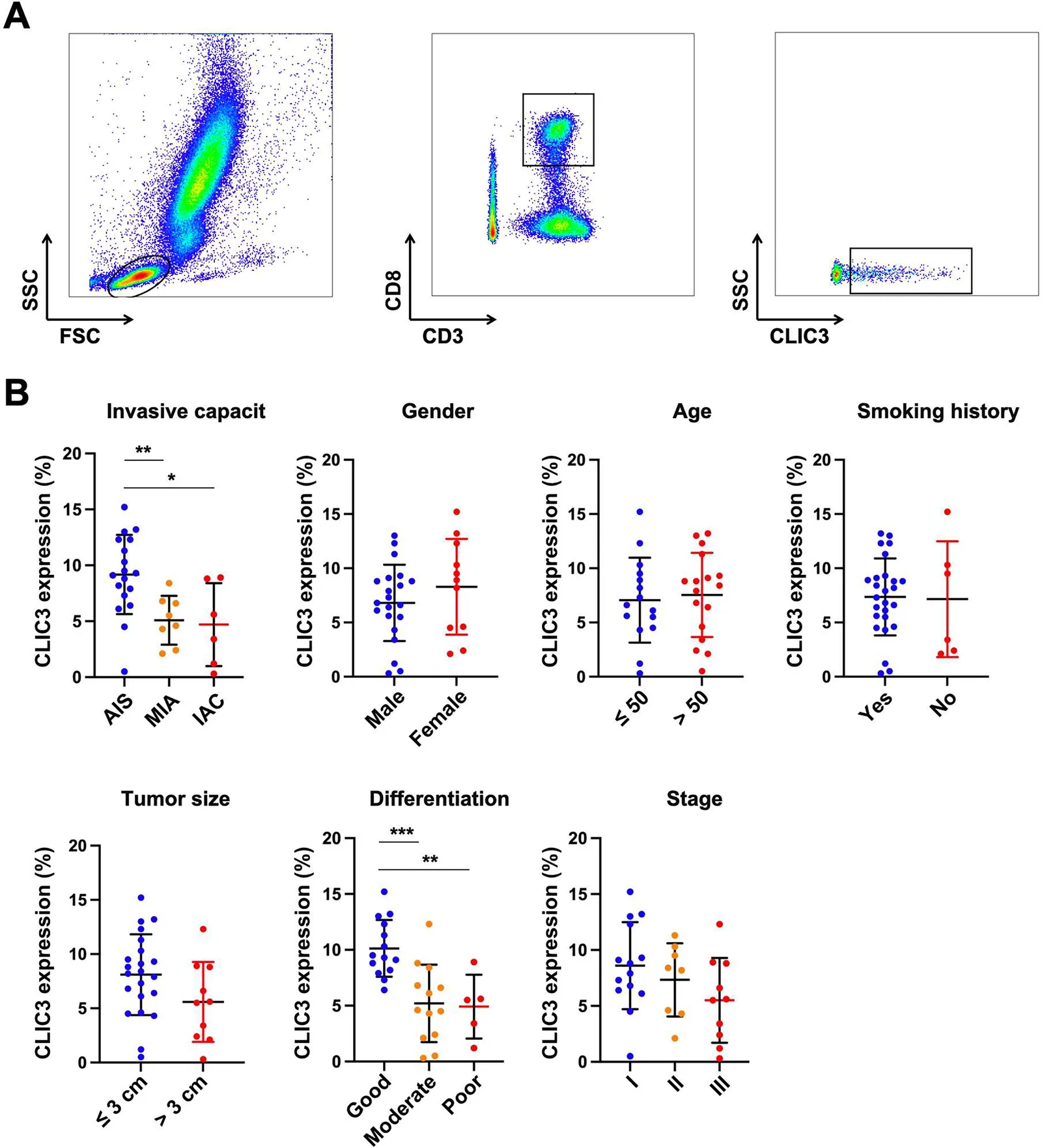
Flow cytometric validation of CLIC3 expression on peripheral blood CD8^+^ T cells in lung adenocarcinoma patients. **(A)** Flow cytometry gating strategy for CLIC3 expression in CD8^+^ T cells. Lymphocytes were gated based on FSC/SSC, followed by selection of CD3^+^CD8^+^ T cells, and finally CLIC3^+^ cells were quantified within the CD8^+^ T cell population. **(B)** Percentage of CLIC3^+^ cells within CD8^+^ T cells stratified by clinicopathological features, including invasive capacity (AIS vs. MIA vs. IAC), differentiation, gender, age, and smoking history. CLIC3 expression decreases with increasing invasive capacity and poor differentiation. No significant differences were observed in gender, age, or smoking history. Data are presented as mean ± SD. ns, not significant; *P < 0.05; **P < 0.01;***P < 0.001.

We next evaluated the association between CLIC3 expression on peripheral blood CD8^+^ T cells and clinicopathological features. Notably, the percentage of CLIC3^+^ cells within CD8^+^ T cells decreased with increasing invasive capacity (**Figure 6B**), showing a directionally consistent trend with our scRNA-seq and spatial transcriptomics findings in tumor-infiltrating T cells. Similarly, patients with poorly differentiated tumors exhibited lower CLIC3 expression compared with those with well or moderately differentiated tumors (**Figure 6B**). In contrast, no significant differences were detected across other clinical parameters, including gender, age, or smoking history; however, given the modest size of this cohort, these subgroup comparisons were likely underpowered, and the absence of significant differences should not be interpreted as evidence that no differences exist. Although not statistically significant, we observed a trend toward lower CLIC3 expression in patients with larger tumor size and more advanced tumor stage. Taken together, these findings indicate that CLIC3 expression on peripheral blood CD8^+^ T cells shows a directionally consistent decline with increasing tumor aggressiveness, although the peripheral blood and tumor compartments are not equivalent, and paired analysis of tumor tissue and peripheral blood will be required to determine whether CLIC3 changes are concordant between the two compartments.

## 4 Discussion

In this study, we systematically characterized the expression pattern and functional significance of CLIC3 in the tumor microenvironment of LUAD across the pathological spectrum from AIS to MIA to IAC. Through an integrative approach combining single-cell RNA sequencing, spatial transcriptomics, bulk RNA-seq analysis, and flow cytometric validation, we identified CLIC3 as a novel marker of CTL functional competence that is lost during LUAD progression. Our findings reveal that CLIC3 is predominantly expressed in CTLs, and its downregulation is associated with the deterioration of effector functions, impaired TCR signaling, and the acquisition of an exhausted phenotype. These results position CLIC3 as a critical molecular determinant of CTL functional integrity within the LUAD microenvironment and suggest its potential as a prognostic biomarker and therapeutic target.

The downregulation of CLIC3 in CTLs from AIS to MIA to IAC represents one of our most compelling findings. This stage-dependent decline suggests that CLIC3 loss is an early event in the immune dysfunction associated with LUAD progression. Interestingly, while the proportion of T cells within the TME actually increased with tumor invasiveness—from 32.9% in AIS to 43.4% in IAC—the functional quality of these T cells, as reflected by CLIC3 expression, deteriorated significantly. This apparent paradox underscores a critical concept in tumor immunology: the quantity of tumor-infiltrating lymphocytes does not necessarily correlate with their functional competence. Our findings align with the broader understanding that tumors can evade immune surveillance not by excluding T cells, but by rendering them dysfunctional through chronic antigen stimulation and immunosuppressive signals within the TME (14, 15). The loss of CLIC3 in CTLs may thus represent a molecular signature of this functional deterioration, providing a more accurate reflection of the anti-tumor immune status than T cell abundance alone.

The functional module scoring analysis provided further insight into the biological significance of CLIC3 expression in CTLs. CLIC3^+^ CTLs exhibited significantly higher scores across all four functional categories examined cytotoxicity, cytoskeleton remodeling, interferon signaling, and chemotaxis compared to their CLIC3 counterparts. This comprehensive functional superiority suggests that CLIC3 expression marks a CTL population with intact effector machinery, cytoskeletal integrity, and migratory capacity. The association between CLIC3 and cytoskeleton remodeling is particularly noteworthy, as CLIC3 has been previously shown to interact with actin cytoskeleton regulators and influence cell migration (10). In T cells, cytoskeletal dynamics are essential for immune synapse formation, TCR signaling, and cytotoxic granule release (16). These processes are integrated within a broader framework of T cell activation, in which TCR signaling, co-stimulatory and co-inhibitory cascades, and cytokine-mediated cues act in concert to determine T cell fate and effector function (17). Perturbations at any of these levels can contribute to functional impairment, and the coordinated decline of CLIC3 with cytoskeletal and signaling-related genes observed here is consistent with such a multi-layered model of T cell dysfunction. The coordinated decline of CLIC3 with cytoskeletal genes such as EZR and MSN in CTLs suggests that CLIC3 loss may compromise the structural integrity required for effective T cell function. Furthermore, the enrichment of interferon signaling and chemotaxis pathways in CLIC3^+^ CTLs indicates that these cells are not only capable of direct cytotoxicity but also possess the ability to recruit and orchestrate broader immune responses within the TME.

The pseudotime trajectory analysis indicated that CLIC3^+^ CTLs are predominantly positioned at one end of the inferred transcriptional continuum, while CLIC3^-^ cells are positioned at the other. Given the cross-sectional nature of the data, this distribution should be interpreted as an inferred ordering rather than a true temporal sequence. Nevertheless, it suggests that CLIC3 expression is associated with a functionally active CTL state, whereas CLIC3 loss is associated with a state characterized by reduced effector features. The expression patterns of key functional genes along the inferred trajectory further supported this interpretation. CD247 (a critical TCR signaling component), GZMB and PRF1 (cytotoxic effector molecules), and TNF (a pro-inflammatory cytokine) all exhibited a decline along the trajectory, paralleling the distribution pattern of CLIC3. This coordinated decline suggests that CLIC3 loss is part of a broader transcriptional program associated with reduced effector function, TCR signaling capacity, and synaptic integrity. Notably, while TOX, a well-established exhaustion-associated transcription factor, remained relatively constant along the trajectory, the decline of CD247 and effector molecules is consistent with a loss of T cell functionality that does not necessarily reach full exhaustion (18). This pattern raises the possibility that CLIC3 loss may represent an early feature of T cell dysfunction that precedes the full exhaustion program, although this hypothesis requires validation in longitudinal or functional studies before it can be interpreted as a temporal sequence. It is also important to distinguish T cell dysfunction from senescence and exhaustion, as these represent mechanistically distinct states with different functional consequences. For instance, DNMT1 deficiency has been shown to induce T cell senescence through altered CDKN1A methylation, leading to impaired effector differentiation independently of the classical exhaustion program (19). In our data, the decline of CD247 and effector molecules without a corresponding increase in TOX suggests that CLIC3 loss may reflect an early dysfunction state that is distinct from both full exhaustion and classical senescence. Whether CLIC3^-^ CTLs acquire senescence-associated features, such as CDKN1A upregulation or senescence-associated secretory phenotypes, remains to be determined and warrants further investigation.

The clinical relevance of our findings was supported by the observation that high CLIC3 expression in the TCGA-LUAD cohort was associated with significantly longer overall survival, despite the lack of correlation with conventional clinicopathological parameters. This prognostic advantage suggests that CLIC3 expression captures a distinct dimension of tumor immune interaction not reflected by standard clinical staging. However, since the TCGA-LUAD dataset is derived from bulk tumor tissues, the CLIC3 signal represents a composite contribution from multiple cell types. The survival benefit therefore cannot be attributed solely to CTL-derived CLIC3; a more measured interpretation is that high CLIC3 expression may reflect a favorable overall microenvironmental state that includes, but is not limited to, functionally competent CTLs. In addition, the cancer type specific expression patterns of CLIC3 upregulated in tumors compared to normal tissues in certain cancer types such as BLCA and CESC, but downregulated in LUAD, LUSC, and HNSC further emphasize the context-dependent nature of CLIC3 biology. This dichotomy may reflect the different cellular origins and TME compositions across cancer types, and underscores the importance of interpreting CLIC3 expression in its specific tissue context. Consistent with pan-cancer biomarker studies such as that by Xiao et al., in which mPRα/PAQR7 was associated with poor prognosis in LUSC, we found that high CLIC3 expression was associated with favorable overall survival in LUAD, reinforcing the context-dependent prognostic significance of molecular markers across cancer types (20).

The tissue expression landscape analysis revealed that CLIC3 is broadly expressed across multiple cell types in the lung, with the highest expression in alveolar type 1 cells, followed by T cells and fibroblasts. This multi-cellular expression pattern likely explains why the GO and KEGG enrichment analyses using TCGA bulk RNA-seq data yielded pathways largely associated with general cellular metabolism, structural processes, and subcellular organelle functions rather than immune-related functions. The abundant expression of CLIC3 in alveolar epithelial cells means that the bulk transcriptomic signal reflects the composite contribution of both immune and non-immune cell populations, potentially diluting the immune-specific signals that were clearly evident in our single-cell analysis. This highlights the power of single-cell approaches in resolving cell-type-specific biological processes that may be obscured in bulk analyses. The nuclear localization of CLIC3 with a punctate distribution pattern, resembling nuclear speckles or splicing bodies, suggests potential roles in RNA processing or transcriptional regulation that warrant further investigation. Notably, CLIC3 lacks the classical nuclear localization sequence found in CLIC1 and CLIC4, and its nuclear entry may depend on association with nuclear-translocated proteins such as ERK7 (21, 22), while its interaction with NAT10 further supports a possible involvement in nuclear RNA-related processes (23). Whether CLIC3 functions as a transcriptional regulator in CTLs, potentially through interaction with nuclear proteins or participation in chromatin remodeling, and how this nuclear pool relates to the transcriptional differences between CLIC3^+^ and CLIC3^-^ CTLs, remain to be determined.

The flow cytometric validation in an independent cohort of 32 LUAD patients confirmed the decline of CLIC3 expression on CD8^+^ T cells with increasing tumor invasiveness and poor differentiation. This protein-level validation bridges the gap between transcriptomic findings and potential clinical application. The trend toward lower CLIC3 expression in patients with larger tumor size and more advanced tumor stage, although not statistically significant, is consistent with our overall findings and may reach significance with a larger sample size. The lack of association with gender, age, or smoking history suggests that CLIC3 expression on CD8^+^ T cells is primarily driven by tumor-intrinsic factors rather than host-related variables, further supporting its role as a tumor-specific immune biomarker. Beyond oncology, the potential value of readily accessible peripheral blood immune parameters as biomarkers has been demonstrated beyond oncology; for example, Yao et al. reported that elevated neutrophil, lymphocyte, and platelet counts were associated with increased preeclampsia risk (24). This broader context supports the notion that peripheral blood CLIC3 expression on CD8^+^ T cells may similarly serve as an accessible biomarker of immune status, although its specificity and predictive value require validation in larger prospective cohorts.

Several limitations of this study should be acknowledged, and potential strategies to address them may inform future research. First, the single-cell RNA sequencing cohort was limited to nine patients (three per pathological stage), and although the findings were validated in an independent flow cytometry cohort, larger-scale multicenter validation studies are warranted to strengthen the statistical robustness and generalizability of the results. Second, the cross-sectional design precludes assessment of the temporal dynamics of CLIC3 expression during tumor progression in individual patients; longitudinal sampling with follow-up data would allow the trajectory of CLIC3 expression on CD8^+^ T cells to be tracked over time and clarify whether CLIC3 loss precedes or follows functional deterioration. Third, while we demonstrated an association between CLIC3 expression and T cell functional competence, the precise molecular mechanisms by which CLIC3 regulates CTL function remain to be elucidated. Future studies employing functional experiments, such as CLIC3 overexpression or knockdown in primary T cells or T cell lines, are needed to establish causality and to identify the downstream signaling pathways involved. Fourth, since CLIC3 is broadly expressed across multiple lung cell types, including alveolar type 1 cells, the CLIC3 correlated genes derived from bulk TCGA data may be substantially influenced by non-immune cell populations; purifying CTLs from tumor tissues and peripheral blood followed by RNA sequencing of these sorted populations would enable a cell type specific dissection of CLIC3-associated transcriptional programs and interaction networks. Fifth, the TCGA pan-cancer analysis revealed that CLIC3 expression patterns are cancer type-specific, suggesting that our findings in LUAD may not be generalizable to other cancer types, and future comparative studies across multiple tumor types will be necessary to define the context-dependent roles of CLIC3. Finally, while we observed a survival benefit associated with high CLIC3 expression, the lack of correlation with conventional clinicopathological parameters suggests that CLIC3 may capture unique aspects of tumor immune interaction that warrant further investigation, ideally in prospective cohorts with standardized treatment and follow-up protocols. Addressing these limitations in future work will be essential for translating our findings into clinically meaningful applications.

In conclusion, this study identifies CLIC3 as a novel marker of CTL functional competence in LUAD that is lost during tumor progression. CLIC3^+^ CTLs exhibit enhanced effector functions, cytoskeletal integrity, and migratory capacity, while CLIC3 loss is accompanied by the decline of TCR signaling components and effector molecules, consistent with the transition toward an exhausted or dysfunctional state. Our findings suggest that CLIC3 expression in CD8^+^ T cells may serve as a predictive biomarker for immunotherapeutic response and a potential target for restoring T cell function in LUAD. In this regard, strategies that target intracellular regulators to restore CD8^+^ T cell killing have shown encouraging results in other settings; for example, inhibition of USP14 was reported to reinstate MIC-A/B-dependent, antigen-independent CD8^+^ T cell killing and to synergize with PD-1 blockade (25). Whether modulating CLIC3 expression could similarly enhance CTL effector function and cooperate with immune checkpoint inhibitors remains an open question, and functional studies combining CLIC3 manipulation with checkpoint blockade will be an important direction for future work. Future research should focus on elucidating the mechanistic basis of CLIC3-mediated T cell regulation and exploring its therapeutic potential in combination with immune checkpoint inhibitors.

## Data Availability

The datasets presented in this study can be found in online repositories. The names of the repository/repositories and accession number(s) can be found in the article/**Supplementary Material**.

## Glossary

AIS: adenocarcinoma in situ
ACN: Acinar Cell Neoplasms
ANOVA: one-way analysis of variance
BLCA: bladder urothelial carcinoma
BP: biological process
CC: cellular component
CESC: cervical squamous cell carcinoma
CLIC: chloride intracellular channel
CMSN: Cystic, Mucinous and Serous Neoplasms
CSCO: Chinese Society of Clinical Oncology
CTLs: cytotoxic T lymphocytes
DAVID: Database for Annotation, Visualization, and Integrated Discovery
DEGs: Differentially expressed genes
FMO: fluorescence-minus-one
FSC: forward scatter
GAMs: generalized additive models
GEO: Gene Expression Omnibus
GO: Gene Ontology
HNSC: head and neck squamous cell carcinoma
IAC: invasive adenocarcinoma
IFN: interferon
IHC: immunohistochemistry
KEGG: Kyoto Encyclopedia of Genes and Genomes
LUAD: lung adenocarcinoma
LUSC: lung squamous cell carcinoma
MIA: minimally invasive adenocarcinoma
MF: molecular function
PBMC: Peripheral blood mononuclear cells
PCA: principal component analysis
ROS: reactive oxygen species
scRNA-seq: single-cell RNA sequencing
SD: standard deviation
SSC: side scatter
ST: spatial transcriptomics
TCGA: The Cancer Genome Atlas
TCR: T cell receptor
TGM2: transglutaminase 2
TSNE: T-distributed stochastic neighbor embedding
TME: tumor microenvironment
UMAP: Uniform Manifold Approximation and Projection
UMI: unique molecular identifier.
