# Supplementary material for: Loss of CLIC3 in CD8+ cytotoxic T lymphocytes is associated with immune dysfunction and poor prognosis in lung adenocarcinoma
Source: Front Oncol. 2026 Sep 30;16:1975159. doi: 10.3389/fonc.2026.1975159 (PMC13662009; doi:10.3389/fonc.2026.1975159)
Supplement: Supplementary file 1 [file Table1.docx]

Table 1. Baseline clinicopathological characteristics of patients with lung adenocarcinoma

| Fatcor | Category |  | CLIC3 high | CLIC3 low |
| --- | --- | --- | --- | --- |
| Age, years |  | 54 (48, 64) | 55 (48, 65) | 50 (46,60) |
| Sex | Male | 16 | 19 | 5 |
|  | Female | 8 | 3 | 3 |
| Smoking history | Yes | 26 | 20 | 6 |
|  | No | 8 | 4 | 2 |
| Tumor size | ≤ 3 cm | 22 | 18 | 4 |
|  | >3 cm | 10 | 6 | 4 |
| Histopathology | well differentiated | 14 | 14 | 0 |
|  | moderately differentiated | 13 | 8 | 5 |
|  | poorly differentiated | 5 | 2 | 3 |
| Stage | I-II | 22 | 17 | 5 |
|  | III-IV | 10 | 7 | 3 |
| Invasive capacit | AIS | 18 | 16 | 2 |
|  | MIA | 8 | 4 | 4 |
|  | IAC | 6 | 4 | 2 |
